# Can substance abuse media literacy increase prediction of drug use in students?

**DOI:** 10.1186/s40359-022-00860-2

**Published:** 2022-06-10

**Authors:** Majid Barati, Saeed Bashirian, Hanieh Jormand, Mohammad Babamiri, Forouzan Rezapur-Shahkolai

**Affiliations:** 1 Health Education and Promotion, Department of Public Health, School of Health and Autism Spectrum Disorders Research Center, Hamadan University of Medical Sciences, Hamadan, IR Iran; 2 Health Education and Promotion, Autism Spectrum Disorders Research Center and Clinical Research Development Unit of Shahid Beheshti Hospital, Hamadan University of Medical Sciences, Hamadan, IR Iran; 3 Department of Ergonomics, School of Public Health and Research Center for Health Sciences, School of Public Health, Hamadan University of Medical Sciences, Hamadan, Iran; 4 Health Promotion, Department of Public Health, School of Public Health and Research Center for Health Sciences, Hamadan University of Medical Sciences, Hamadan, Iran

**Keywords:** Substance use, Communications media, Students, Prototype willingness model

## Abstract

**Objective:**

The present study aimed to determine the predictors of drug use among students based on the Extended Prototype willingness model (EPWM).

**Methods:**

This cross-sectional study was performed at Hamadan universities. The participants were selected via multi-stage sampling. Finally, out of 721 students participated. The questionnaire consisted of four sections: (a) socio-demographic characteristics, (b) Questionnaire about the prototype willingness model constructs, (c) Questionnaire about the status of drug use behavior, (d) Substance Abuse Media Literacy (SAML) scale. Data were analyzed using a structural equations partial least squares confirmatory composite approach and SPSS-23.

**Results:**

The mean age of students was of 23.47 ± 4.14 years. Based on results, behavioral willingness (β = 0.420, t-value = 9.010, *p* < 0.001) and behavioral intention (β = 0.291, t-value = 6.957, *p* < 0.001) significantly predicted drug use. The presented results of analysis, 66% of the variance of the drug use, 62% of the variable of the behavioral willingness, and 56% of the behavioral intention could be explained by the EPWM.

**Conclusion:**

The present study demonstrated that EPWM could predict drug use in students. Therefore, designing and implementing educational and promotional intervention programs to reduce behavioral willingness and increase students’ skills in substance abuse media literacy is necessary to prevent drug use behavior.

## Introduction

According to the United Nations Office on Drugs and Crime 2020 (UNODC), there were about 269 million (range: 166–373 million) people who used substances worldwide in 2018, which is, unfortunately, a 30% increase over 2009 [1]. This latest report from the office also mentioned the existence of 35 million people who use drugs [2]. In addition, a study by the United Nations Office on Drugs and Crime 2019 found that the prevalence of opium increased in Africa, Asia, Europe, and North America, and cannabis use in North and South America and Asia. Cannabis and marijuana, with 188 million users, are the most common drugs in the world, with increasing use in the United States and Asia [2]. Deaths from used drugs amounted to 450,000 deaths, of which 16,750 were estimated to be due to drug overdose [1, 3]. This problem is one of the most important social, political, and health challenges in various countries, including Iran. A study of the global burden of diseases in Iran in 2016 showed that the rate of disability due to drug use a per 100,000 people reached 521, and deaths due to drug use per 100,000 people had reached 2.7, which is compared to the rate of disability due to used drugs in the world is 31.8 million disabilities which are increasing [4].

Due to its young population and geographical location, Iran is mainly exposed to this global problem [2, 5]; despite legal efforts in the country to prevent substance use behavior, this has been progressive among Iranian youth in recent years [6, 7].

Evidence suggests that risky behaviors such as smoking, use of alcohol, and drug use often begin in late adolescence and youth, leading to increased mortality, morbidity, and major public health challenges [8]. Accordingly, students can be considered as one of the high-risk groups for using drugs [9].

The results of another study showed the predictors of behavioral intention in drug use among students such as attitudes, subjective norms, and risk images [10], low level of knowledge, living in places without family supervision, male gender, high level of income [11]. Furthermore, the results of some studies also showed that new media such as websites and social networks apps are considered the most important environmental risk factor for drug use, especially among young people [12–15].

### Media literacy and substance abuse media literacy skills

Media literacy is the cognitive process utilized in critical thinking [16]. With media literacy, an individual can cope, manage their own media activity, and intentionally choose the media and information they receive [17]. The wide range of media messages, risks, and harms in cyberspace make media literacy skills essential for critically analyzing media messages [14, 18].

Media techniques and persuasion, highlighting initial pleasant consequences, claims of harmlessness, and tricks by media content encouraging substance abuse to attract and persuade the audience to pursue advertising and even encouraging substance abuse [15]. With the appearance of new media technology, and new concepts such as critical thinking about creating/producing, creator and sharing media content in various channels, political-economic purpose of media massages emission and most importantly critical thinking about methods of persuading the audience by creators of media massages, and presenting new patterns in lifestyle by misinformation and fake news thorough consuming media skills in individuals were introduced (Koc and Barut 2016). Austin et al. evidenced that young people and adolescents were addressed as a young and inexperienced class with low cognitive growth by the media encouraging substance abuse (Austin and Pinkleton 2016. So, acquiring specific media literacy skills such as substance abuse media literacy skills, especially for young people, is mainly essential.

Also, meta-analysis studies regarding the effectiveness of media literacy interventions on young people’s high-risk behaviors suggested that media literacy educational interventions effectively prevent drug use in youth and adolescents [19, 20].

## Theoretical framework

Concerning all predictors and constructs mentioned, the prototype willingness model (PWM) was appropriate for this study. In this regard, Gerald and Gibbons seek to investigate unintentional behaviors, especially during adolescence and youth, using approaches that take the decision process away from the rational path; they recommend this process as an exploratory process and believe that young people use mental imagery such as social reaction to make their decisions.

Now, where did these mental imagery or prototype come from? When this issue is addressed among adolescents and young adults, it will be the most common response on television and in movies, followed by magazines, family, peers, and friends. The influence of the medium on prototype and willingness has also been reported in examining the association between exposure to substance use content movies and substance use in adolescents and young adults [21]. In addition, the results of a systematic review and meta-analysis study showed that exposure to persuasive media leads to a 27% increase in adolescent smoking intention; also, media such as film, as the most vital media encouraging smoking, increase their smoking by 54% [14]. So, regarding the role of prototypes and unintentional behavior of adolescents and adults, the PMW was suitable for the present study.

It should be noted that contrary to the theory of logical action, such as the Theory of Reasoned Action, Theory of Planned Behavior, etc., in which past behavior does not affect behavioral intention, the PWM of past behavior is also considered [22]. According to the theory of rational action, decision-making is a logical and planned process considering the predicted results of behavior. When making decisions to perform the behavior, attitudes through behavioral intention affect behavior [23]. In addition, a study conducted with the theory of planning behavior showed that intentional behaviors typically explain 30 to 40% of the variance of behavior [24, 25], especially in healthy behaviors [26]. However this theory has little role in predicting some behaviors, especially those with lower social acceptance, mainly when used to predict high-risk behaviors such as alcohol consumption or high-risk sexual behaviors targeted at young people have been less successful [27].

**Fig. 1:**
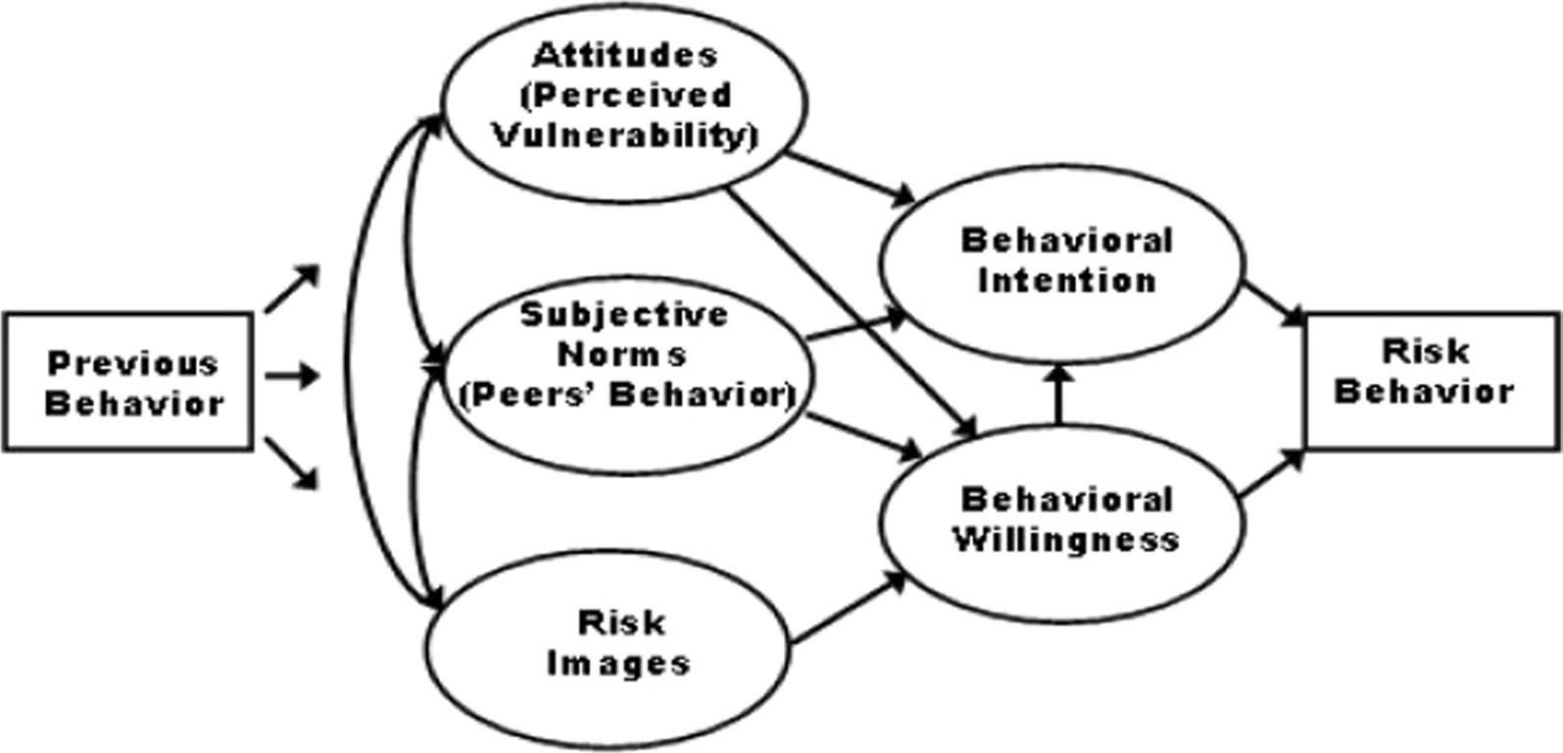
The framework of the prototype willingness model (PWM) [27]

Evidence suggests the function of the PWM to predict high-risk behaviors in youth and adolescents [28]; In other words, numerous studies have already been done about the predictors of drug use among college students [29–31] (Fig. 1).

On the other hand, the role of new media as the most important environmental risk factor for using drugs in the high-risk group [13, 14, 32] and the essential role of acquiring media literacy, substance abuse media literacy (SAML) variable [33], as a background variable added to the PWM model; and model extended Fig. 2.

**Fig. 2:**
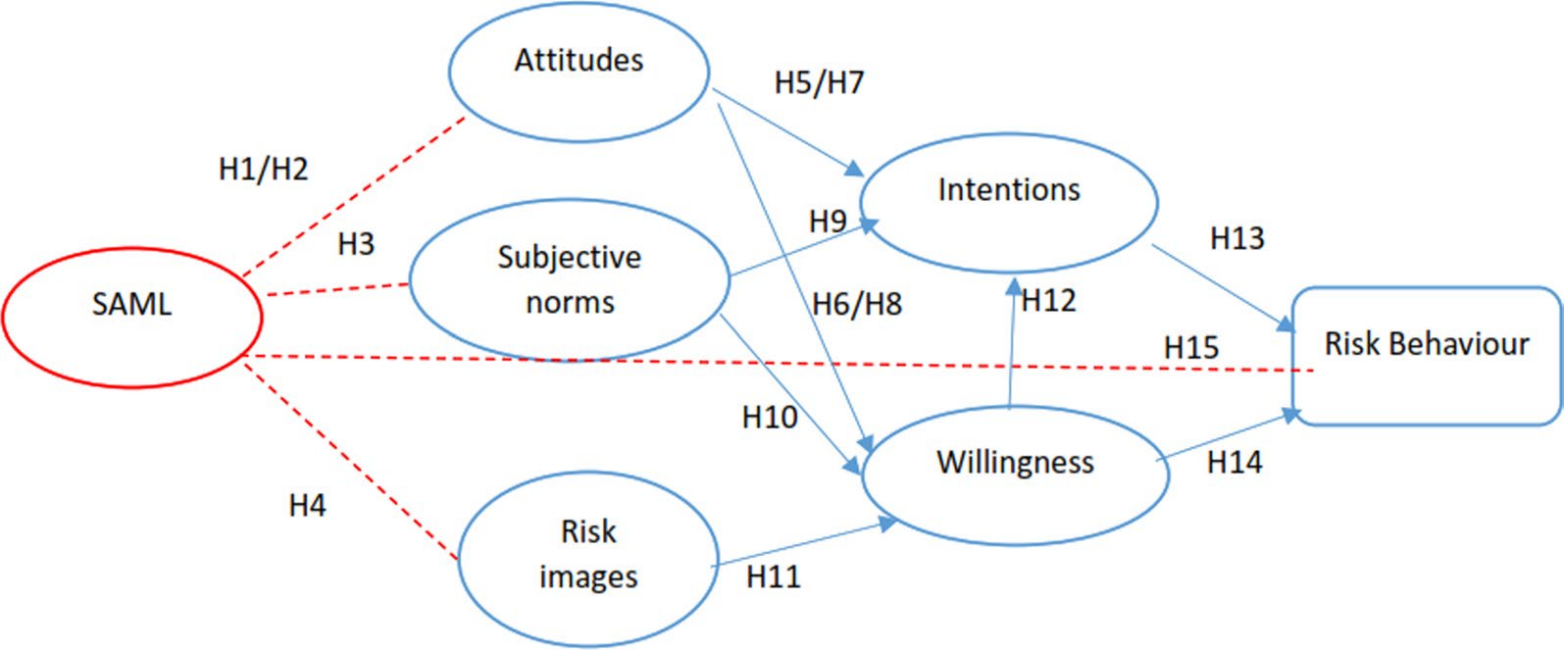
The framework of the extended prototype willingness model (EPWM)

Therefore, the present study aimed to determine the predictors of drug use among university students in Hamadan based on the EPWM model. Hence, this research hypothesizes:

- *H1*. The SAML will have a negative and significant effect on their positive attitude towards drug use.
- *H2*. The SAML will have a positive and significant effect on their negative attitude towards drug use.
- *H3*. The SAML will have a negative and significant effect on their subjective norms.
- *H4*. The SAML will have a negative and significant effect on their risk images.
- *H5*. A positive attitude towards drug use will have a positive and significant effect on their intention to drug use.
- *H6*. A positive attitude towards drug use will have a positive and significant effect on their willingness towards drug use.
- *H7*. The negative attitude towards drug use will have a negative and significant effect on their intention to drug use.
- *H8*. The negative attitude towards drug use will have a negative and significant effect on their willingness towards drug use.
- *H9*. The subjective norms will have a positive and significant effect on their intention to drug use.
- *H10*. The subjective norms will have a positive and significant effect on their willingness toward drug use.
- *H11*. The drug use images will have a positive and significant effect on their willingness towards drug use.
- *H12*. The willingness towards drug use will have a positive and significant effect on their intention to drug use.
- *H13*. The intention to drug use will have a positive and significant effect on their drug use behavior (high-risk behavior).
- *H14*. The willingness towards drug use will have a positive and significant effect on their drug use behavior.
- *H15*. The SAML will have a negative and significant effect on their drug use behavior (high-risk behavior).

Figure 2 illustrates the hypothesized relationships of this research.

## Methods

### Design and participants

This cross-sectional descriptive study was done on 721 students of Universities in Hamadan, Iran who were recruited through a stratified random sampling procedure between May and June 2020. Hamadan City is located in the west of Iran. It has four state universities, including Bu-Ali Sina University, Hamadan University of Medical Sciences, Hamadan University of Applied Sciences & Technology, and the Hamadan University of Technology. Moreover, it has some non-governmental universities, such as Islamic Azad University Hamadan Branch and Hamadan Payam Noor University [34]. Therefore, the number of students in each school of the University was obtained by proportional assignment of students to the school (i.e., the school with the largest number of students, the largest number of students assigned) after coordinating with university officials and the research units were rationed. Then, the samples were randomly collected. The word informed consent was obtained from all students; they were informed about the confidentiality of the information and the project’s purpose, and only if they would like they were enrolled in the study. The Ethics Committee approved this study with all consent processes at Hamadan University of Medical Sciences.

### Data collection and measurements

Data collection tools included a researcher-made four-part questionnaire, socio-demographic questions, PMW structures based on PWM-based literature, and studies [29, 35] status of drug use behavior and substance abuse media literacy (SAML) questions.

Demographic information questionnaire consists of (age, gender, marital status, the field of study and educational status, parents’ residence status, education, and the status of using social networks apps and computers, and motivation to use social networks apps with ten questions.

Also, ten questions about the status of substance use as a high-risk behavior checklist were used to collect data on their use of cigarettes and the type of substance and other drugs in him/ the form of three-choice questions (never, sometimes, and always) [11].

Besides, PWM constructs were assessed using the PWM scale for drug use risk behaviors. It was compiled of 21 items and seven subscales as follows:

Positive attitude toward drug use (7 items, e.g., “If I take drugs, it will help me to increase my concentration.”) scored on a 5-point Likert scale with answers ranging from ‘strongly disagree’ to ‘strongly agree.’ The scores of this construction ranged from 7 to 35. The highest score in this section indicated a positive attitude toward drug use.

Negative attitude toward drug use (3 items, e.g., “If I take drugs, I will waste my money.”) scored on a 5-point Likert scale with answers ranging from ‘strongly disagree’ to ‘strongly agree.’ The scores of this construction ranged from 3 to 15. The highest score in this section indicated a positive attitude towards drug use.

Subjective norms focused on normative beliefs and motivation to comply (2 items, e.g., normative beliefs: “My best friend’s opinion about drugs is that I am….…”; which were rated on a 5-point scale ‘I should not use at all to ‘I must consume’; 2 other items about motivation to comply scored on a 5-point Likert scale "I think my best friend views, I have to accept drugs” with answers ranging from ‘strongly disagree’ to ‘strongly agree’. The Subjective norms structure scores ranged from 4 to 20 [36].

Prototypes about drug use presented positive drug user images and non-drug user images (7 items in terms of inexperienced and naive, self-confidence, independence, attractiveness, and selfishness. This construct was rated on a 5-point Likert scale from ‘very much’ to ‘never. A higher score indicated the subject’s positive prototype towards drug use student/healthy student.

Behavioral willingness includes a scenario in which a student thinks that he/she is among her friends who use drugs and persuade her/him to drug use. In this section, the student’s feedback to this scenario was evaluated following four feedbacks: (1) I take the drugs and use one or more of them; (2) I use drugs with my friends until the end; (3) I thank them and I refuse to drug use, and (4) I leave that place. The answers were measured on a 5-point Likert scale ranging from ‘never’ to ‘very much. The scores ranged from 4 to 20, respectively. A higher score indicated a greater willingness for drug use [36].

Behavioral intention to drug use with two questions which show the intention to drug use in the next month and then 6 months later (e.g.: ‘I may use drugs in the next month’). The scores ranged from 2 to 10, with a higher score indicating a student’s higher intention to drug use.

Also, the question about the status of drug users /herself was used with yes/no questions [11].

In section four, substance abuse media literacy (SAML) in students was evaluated with 13 questions and scored on a 5-point Likert scale with answers ranging from ‘strongly disagree’ to ‘strongly agree.’ The scores of this construction ranged from 13 to 65. The highest score in this section indicated higher media literacy related to substance use [33]. It is essential to be mentioned that there are several general and specific tools to measure media literacy. Each of these tools has introduced dimensions for media literacy. These tools suffer from their narrow scopes and lack of any instrument targeted at measuring media literacy in specific issues to assess the medial literacy holistically in terms of multidimensional skill. Moreover, the multidimensional specific media literacy measure SAML has been mainly neglected. Although some studies have been conducted using the researcher-made questionnaire about media literacy in Iran [9, 37, 38].

Hence, Based on the previous study, the SAML scale, which included 13 items, was evaluated, was a valid and reliable tool, and now is suitable and acceptable for present studies [33]. Also, this scale was supported in previous research [39–41] and developed based on the qualitative analysis of the exploration of substance abuse media literacy among students [15].

The inclusion criteria were being a student of one of the universities in the city of Hamadan, being interested in participating in the study, ability to respond and take part in the study, and access to the Internet and social networks such as what’s app, telegram, and Instagram and YouTube.

Questionnaires were completed through self-report by students who had informed consent to study.

Structural Equation Modeling (SEM) is a technique used to specify, estimate, and evaluate models of linear models among a set of observed variables in terms of an often smaller number of unobserved variables. SEM may be used to build or test the theory. The SPSS software version 23 and a structural equations partial least squares’ confirmatory composite approach (PLS) version 3 was used to analyze the data. Also, PLS (Partial Least Squares) are less prone to type I error and better suited for small, non-normal datasets often collected for initial tests of relationships. Partial Least Squares, which focuses on the analysis of variance, can be carried out using Smart PLS and WarpPLS, etc. [42].

## Results

### Descriptive statistics

The age range of the study participants was between 17 and 49 years, with a mean of 23.47 ± 4.14 years; According to the findings, 60.9% of the study participants were in the age group of 20 to 25 years. 62.8% of the participants in the study were female, and 87.1% of them were single. 78.1% of the study participants were BS students. 43.1% of participants reported living in dormitories; 73.2% of the students were unemployed.

According to the findings, 28.3% of students had access to a computer for more than ten hours a day compared to previous research, which reported that 27.3% of students suffered from problematic Internet use [43], which is significant and was increased. 40.5% of students had access to and used the Internet for more than 10 h daily, which is significant. 56.3% of study participants sometimes used social network apps for entertainment and fooled around online; also, 62.0% reported using social networks apps for information (Table 1).

**Table 1:** Demographic variables in study participants

Variables	N (%)	Percent
*Age*		
< 20	133	18.4
21–25	439	60.9
26–30	106	14.7
> 30	43	6.0
*Sex*		
Woman	453	62.8
Men	268	38.2
*Marital status*		
Single	628	87.1
Married	93	12.9
*Job*		
Jobless	528	73.2
Employed	193	26.8
*Grade*		
B.S	563	78.1
M.S	92	12.8
Ph.D.	66	9.2
*Living condition*		
With Parent	153	21.2
Dormitory	311	43.1
Suite Student	257	35.6
*Field of study*		
Medical Group	351	48.7
Engineering Group	99	13.7
Humanities Science Group	95	13.2
Basic Science Group	89	12.3
Art Science Group	87	12.1
*Father education*		
Illiterate	34	4.7
Elementary	189	26.2
Diploma	247	34.3
University	288	39.9
*Mather education*		
Illiterate	63	8.7
Elementary	189	26.2
Diploma	292	42.5
University	177	24.5

### Descriptive statistics of items in the extended prototype willingness model

The values obtained for the structures of the extended prototype willingness model (EPWM) among the participants in the study show that among the constructs of the studied model, the substance abuse media literacy (SAML) with 65.56% and negative attitude related to drug use behavior with 69.33% of the average score of the maximum achievable score have the highest frequency and status. It was relatively desirable. It is necessary to explain that this percentage is a kind of correct judgment and the mean alone cannot be judged and how to calculate it as the ratio of the difference between the mean of the minimum score on the range of scores is expressed as a percentage.

While positive attitudes related to drug use, behavioral willingness, and intention were evaluated in an unfavorable situation (Table 2).

**Table 2:** Mean of prototype willingness model variables and substance abuse media literacy

Construct	Mean (SD)	Range	Percentage^a^
Substance abuse media literacy	47.09 ± 9.50	13–65	65.56
Positive attitude	15.36 ± 6.76	7–35	29.86
Negative attitude	11.32 ± 2.89	3–15	69.33
Subjective norms	6.18 ± 2.95	4–20	13.63
Positive drug user prototypes	7.57 ± 3.26	3–15	38.08
Positive non-drug user prototype	10.21 ± 4.14	4–20	38.81
Willingness	7.03 ± 3.97	4–20	18.94
Intention	3.08 ± 2.01	2–10	13.5

^a^Percentage of the mean from the maximum obtainable score

### Confirmatory composite analysis

**Fig. 3:**
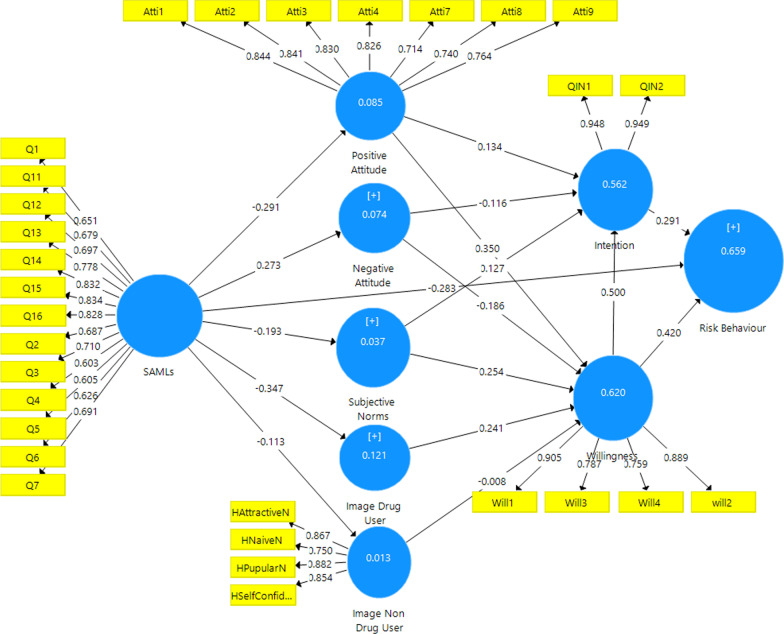
Path coefficient results of EPWM

The standardized loadings values and the standardized factor loading were higher than 0.6 [44, 45]. Also, considering the t-statistic above ± 1.96. Other thresholds of calculated values, such as the composite reliability values and the average variance extracted (AVE) [46] presented in Tables 3, 4 and Fig. 3

**Table 3:** Convergent validity results which assure acceptable values (factor loading > 0.60, Cronbach’s Alpha, composite reliability ≥ 0.70 and AVE > 0.5)

Construct	Items	Outer loadings	Cronbach’s Alpha	CR	AVE
Substance abuse media literacy	Q1	0.651	0.918	0930	0.510
	Q11	0.679			
	Q12	0.697			
	Q13	0.778			
	Q14	0.832			
	Q15	0.834			
	Q16	0.828			
	Q2	0.687			
	Q3	0.710			
	Q4	0.603			
	Q5	0.605			
	Q6	0.626			
	Q7	0.691			
Positive attitute	Atti1	0.844	0.903	0.923	0.633
	Atti2	0.841			
	Atti3	0.830			
	Atti4	0.826			
	Atti7	0.714			
	Atti8	0.740			
	Atti7	0.764			
Negative attitute	Atti5	0.614	0.736	0.843	0.647
	Atti6	0.880			
	Atti10	0.889			
Subjective norms	SN1	0.766	0.770	0852	0.591
	SN2	0.784			
	SN5	0.755			
	SN6	0.769			
Positive drug user prototypes	HNavieD	0.737	0.665	0.812	0.590
	HSelfConfidenceD	0.799			
	HSelfishD	0.767			
Positive non-drug user prototypes	HAttractiveN	0.867	0.865	0.905	0.705
	HNavieN	0.749			
	HPapularN	0.882			
	HSelfConfidenceN	0.855			
Willingness	Will1	0.905	0.857	0.903	0.701
	Will2	0.882			
	Will3	0.786			
	Will4	0.761			
Intention	QINT1	0.948	0.888	0.947	0.900
	QINT2	0.949

**Table 4:** Cross-loading results

	Positive attitude	Negative attitude	Drug user prototypes	Non drug user prototype	Intention	SAMLs	Subjective norms	Willingness
Atti1	**0.844**							
Atti2	**0.841**							
Atti3	**0.830**							
Atti4	**0.826**							
Atti7	**0.714**							
Atti8	**0.740**							
Atti9	**0.764**							
Atti5		**0.615**						
Atti6		**0.879**						
Attti10		**0.889**						
HAttractiveN				**0.867**				
HNaiveD			**0.737**					
HNaiveN				**0.749**				
HPupularN				**0.882**				
HSelfConfidenceD			**0.799**					
HSelfConfidenceN				**0.855**				
HSelfishD			**0.767**					
Q1						**0.651**		
Q11						**0.679**		
Q12						**0.697**		
Q13						**0.778**		
Q14						**0.832**		
Q15						**0.834**		
Q16						**0.828**		
Q2						**0.687**		
Q3						**0.710**		
Q4						**0.603**		
Q5						**0.605**		
Q6						**0.626**		
Q7						**0.691**		
QIN1					**0.948**			
QIN2					**0.949**			
SUB1							**0.766**	
SUB2							**0.784**	
SUB5							**0.755**	
SUB6							**0.769**	
Will1								**0.905**
Will3								**0.786**
Will4								**0.761**
will2								**0.889**

The standardized loadings values and the standardized factor loading were higher than 0.6

Although, the discriminant validity of the construct was calculated as the Fornell–Larcker Scale [47]. The present study also examined the heterotrait-monotrait (HTMT) ratio, which was considered lower than 0.9; thus, it re-confirmed the presence of discriminant validity [46] (Tables 7, 8).

### Assessment of structural model (inner model)

The PLS algorithm revealed the model’s predictive power regarding the coefficient of determination (R^2^) of the endogenous latent variables [46]. Presented the analysis results, the model 56% of the behavioral intention (R square = 0.562), while 66% substance used disorders behavior (R square = 0.659) as the dependent variable can be explained based on EPMW model (Table 5; Fig. 3).

**Table 5:** R2 of the endogenous latent variables

Construct	R square	Result
Positive attitude	0.085	Weak
Negative attitude	0.074	Weak
Subjective norms	0.037	Weak
Drug user prototypes	0.121	Weak
Non drug user prototypes	0.013	Weak
Intention	0.562	Moderate
Willingness	0.620	Moderate
Risk behaviour	0.659	Moderate

### Structural model analysis

H1 to H4 and H15 show that base on result there were association between the SAML (β = − 291, t-value = 8.918, *p* < 0.001) with positive attitude, negative attitude (β = 0.273, t-value = 6.830, *p* < 0.001), subjective norms (β = − 0.193, t-value = 4.332, *p* < 0.001), drug user images (drug user prototypes) (β = − 0.347, t-value = 8.412, *p* < 0.001) and, risk behavior (β = − 0.283, t-value = 10.613, *p* < 0.001). On the other word, Negative association between SAML as background factor with PMW model constructs such as positive attitude, negative attitude, subjective norms, drug user prototypes and, risk behavior (*p* < 0.001).

H5 revealed positive attitude (β = 0.134, t-value = 3.666, *p* < 0.001) was associated with behavioral intention. So, behavioral willingness was the best predictor of behavioral intention rather than other constructs.

**Fig. 4:**
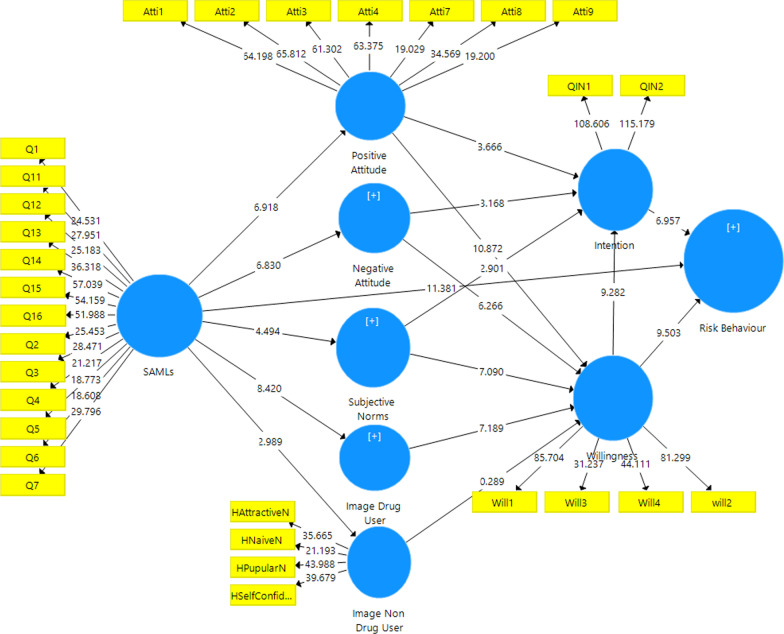
PLS-SEM bootstrapping of EPWM

Moreover, H11, H12, H13, and H14 indicated drug user images (drug user prototypes) were associated with the willingness (*p* < 0.001). Behavioral willingness (β = 0.420, t-value = 9.010, (CI: 0.320, 0.509), *p* < 0.001) and behavioral intention (β = 0.291, t-value = 6.957, *p* < 0.001) significantly predicted risk behavior. So, behavioral willingness was the best predictor of substance use behavior rather than behavioral intention. In other words, 95% are confident that the power predictor of behavioral willingness with 0.42 is between 0.320 and 0.509, while the power predictor of behavioral intention with 0.29. However, the mediating analysis reported full mediation from the willingness to construct the association between intention and risk behavior as β = 0.420, t = 9.010, and *p* < 0.001 (Table 6; Figs. 3, 4).

**Table 6:** Results of structural model

Relationship	Original sample (path)	Sample mean	Confidence intervals bias corrected	f square	t-value	*p* value	Direction	Decision
H1: SAML -> Positive attitute	**−** **0.291**	**−** **0.293**	**[−** **0.370, −** **0.209]**	0.093	**6.918**	**0.000**	**Negative**	Supported**
H2: SAML -> Negative attitute	**0.273**	**0.273**	**[0.192, 0.349]**	0.080	**6.830**	**0.000**	**Positive**	Supported**
H3: SAML -> Subjective norms	**−** **0.193**	**−** **0.194**	**[−** **0.276, 0.345]**	0.039	**4.332**	**0.000**	**Negative**	Supported**
H4: SAML -> Images drug user	**−** **0.347**	**−** **0.350**	**[0.428, −** **0.269]**	0.137	**8.412**	**0.000**	**Negative**	Supported**
H4: SAML -> Images non drug user	**−** **0.113**	**−** **0.116**	**[−** **0.194, −** **0.044]**	0.013	**2.898**	**0.003**	**Negative**	Supported**
H5: Positive attitute -> intention	**0.134**	**0.130**	**[0.055, 0.193]**	0.022	**3.666**	**0.000**	**Positive**	Supported**
H6: Positive attitute -> Willingness	**0.350**	**0.349**	**[0.278, 0.408]**	0.208	**10.872**	**0.000**	**Positive**	Supported**
H7: Negative attitute -> intention	**−** **0.116**	**−** **0.118**	**[−** **0.192, −** **0.052]**	0.022	**3.168**	**0.002**	**Negative**	Supported**
H8: Negative attitute -> Willingness	**−** **0.186**	**−** **0.186**	**[−** **0.246, −** **0.133]**	0.067	**6.266**	**0.000**	**Negative**	Supported**
H9: Subjective norms -> intention	**0.127**	**0.128**	**[0.049, 0.220]**	0.023	**2.901**	**0.004**	**Positive**	Supported**
H10: Subjective norms -> Willingness	**0.254**	**0.258**	**[0.187, 0.334]**	0.120	**7.090**	**0.000**	**Positive**	Supported**
H11: Drug user images -> Willingness	**0.241**	**0.239**	**[0.178, 0.304]**	0.079	**7.189**	**0.000**	**Positive**	Supported**
H11: Non drug user images -> Willingness	**−** **0.008**	**−** **0.007**	**[−** **0.061, 0.048]**	0.000	0.289	0.773	**Negative**	Not supported
H12: Willingness -> intention	**0.500**	**0.500**	**[0.390, 0.611]**	0.238	**9.282**	**0.000**	**Positive**	Supported**
H13: Intention -> Risk behaviour	**0.291**	**0.294**	**[0.199, 0.382]**	0.116	**6.957**	**0.000**	**Positive**	Supported**
H14: Willingness -> Risk behaviour	**0.420**	**0.418**	**[0.320, 0.509]**	0.239	**9.010**	**0.000**	**Positive**	Supported**
H15: SAML -> Risk behaviour	**−** **0.283**	**−** **0.283**	**[−** **0.334, −** **0.231]**	0.201	**10.613**	**0.000**	**Negative**	Supported**

Research hypotheses significant at **p < 0.01

The results indicated significant f^2^ values between behavioral willingness and behavioral intention (f^2^ = 0.238) and between behavioral willingness and risk behavior (f^2^ = 0.239). Also, f2 values between SAML and risk behaviour (f2 = 0.201) (Table 6).

**Fig. 5:**
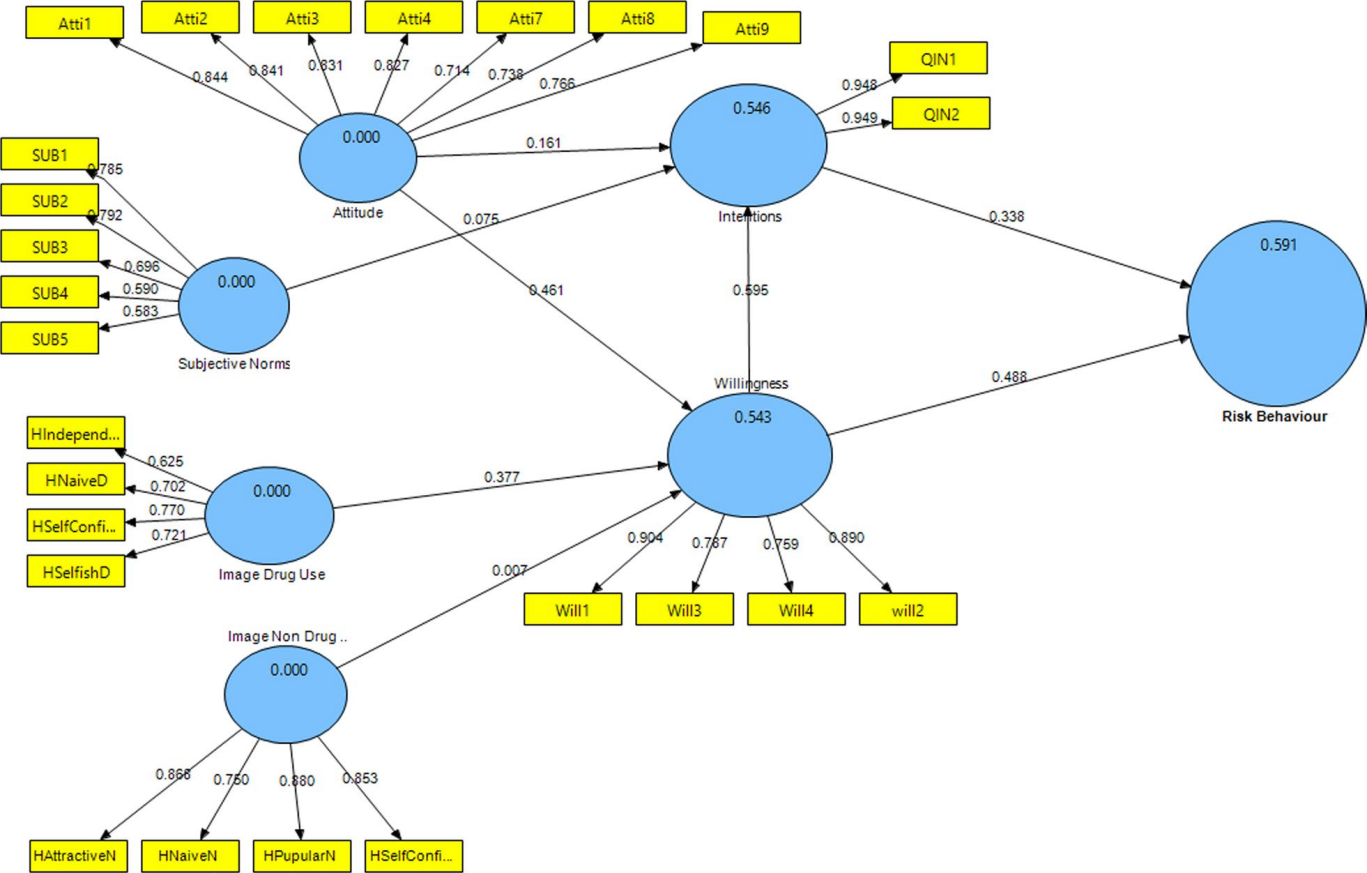
Path coefficient results of PWM

**Fig. 6:**
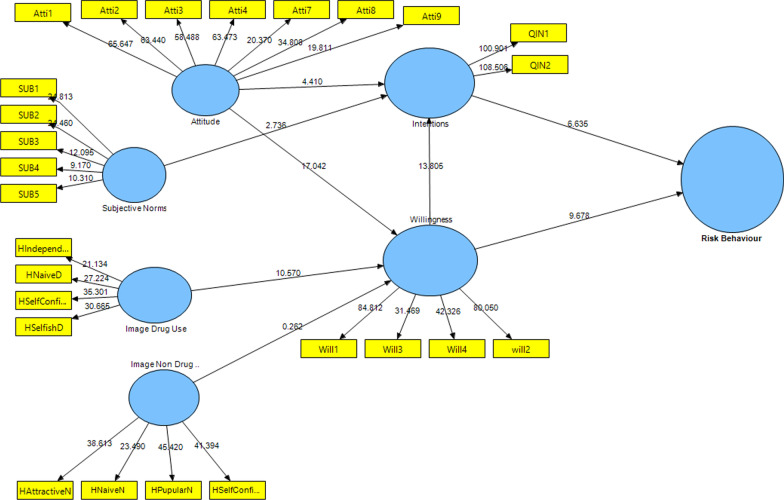
PLS-SEM bootstrapping of PWM

Although, the results showed, based on the PWM, 59% of the variance of the variable behavior of substance use, 55% of the behavioral willingness, and 55% of intention were explainable in Figs. 5 and 6 (Tables 7, 8).

**Table 7:** Fornell–Larcker scale

	Drug user prototypes	Non drug user prototypes	Intention	Negative attitude	Positive attitude	Risk behaviour	SAMLs	Subjective norms	Willingness
Drug user prototypes	0.768								
Non drug user prototypes	0.546	0.840							
Intention	0.578	0.245	0.948						
Negative attitude	**−** 0.448	**−** 0.259	**−** 0.478	0.804					
Positive attitude	0.492	0.252	0.572	**−** 0.385	0.796				
Risk behaviour	0.682	0.202	0.691	**−** 0.522	0.578	1.000			
SAMLs	**−** 0.347	**−** 0.113	**−** 0.338	0.273	**−** 0.291	**−** 0.531	0.714		
Subjective norms	0.400	0.199	0.532	**−** 0.379	0.489	0.439	**−** 0.193	0.769	
Willingness	0.594	0.311	0.725	**−** 0.523	0.663	0.732	**−** 0.356	0.591	0.837

**Table 8:** Heterotrait–Monotrait ratio (HTMT)

	Drug user prototypes	Non-drug user prototypes	Intention	Negative attitude	Positive attitude	Risk behaviour	SAMLs	Subjective norms	Willingness
Drug user prototypes									
Non-drug user prototypes	0.767								
Intention	0.727	0.262							
Negative attitude	0.600	0.290	0.552						
Positive attitude	0.604	0.263	0.636	0.421					
Risk behaviour	0.802	0.197	0.733	0.575	0.604				
SAMLs	0.421	0.118	0.370	0.312	0.316	0.551			
Subjective norms	0.533	0.219	0.640	0.463	0.583	0.499	0.226		
Willingness	0.754	0.341	0.822	0.595	0.753	0.776	0.388	0.723	

Surprisingly, after the addition of the substance abuse media literacy variable, as a background variable to the model; and extended model, the predictive power was improved as 66% of the variance of the risk behavior variable, 62% of the variable of behavioral willingness, and 56% of the behavioral intention could be explained by the extended prototype willingness model (EPWM) (Figs. 3, 5).

## Discussion

The present study was conducted to determine the predictors of drug use among university students in Hamadan based on the EPWM model.

In this regard, Gerald and Gibbons seek to investigate unintentional behaviors, especially in adolescence and youth, and suggest using approaches that take the decision process away from the rational path; they recommend this process as an exploratory process and believe that young people use mental imagery such as social reaction to make their decisions. It should be noted that contrary to the theory of logical action in which past behavior does not affect behavioral intention, the PWM of past behavior is also considered. This model introduces prior behavior as a prelude to a positive attitude toward behavior, subjective norms, behavioral intention, and behavioral willingness [28]. Evidence suggests the function of the PWM to predict high-risk behaviors such as smoking in youth and adolescents [29–31, 48]. Therefore, it is necessary to apply theories and behavioral patterns of dual- processes that, in their psychological analysis, in addition to logical decision-making processes, also pay attention to the decision-making process combined with emotional and behavioral willingness is essential [27, 29, 49].

Based on the findings, 66% of the variance of the drug use as a risk behavior variable, 62% of the variable of the behavioral willingness, and 56% of the behavioral intention could be explained by the EPWM. In line with the study of Rahimi et al., which used the model of PMW to predict the water pipe smoking in high school students, 49% of the variance of behavioral intention and 54% of the variance of smoking behavior by the PWM was predictable [30]. In addition, in Bashirian et al.’s study on predicting shisha smoking in adolescent females students, the PMW was able to predict 74% of the variance of behavior, 70% of the variance of behavioral intention, and 62% of the variance of behavioral willingness in female students [36]. So, these results indicate that the PWM can predict high-risk behaviors in which social reaction to rational action is important [50].

According to the innovation created in the PWM, the prediction power of this model has increased by adding substance abuse media literacy and an extended model to EPWM. Therefore acquiring media literacy skills related to drug use and empowering students along with designing and performing interventions to improve commonly known variables as risk factors of drug use such as attitude, subjective norms, prototypes, behavioral willingness, and behavioral intention to drug use might help to reduce this high-risk behavior in youth. It should be noted that the research of Primack et al. emphasized acquiring media literacy skills and reducing the risk of smoking in students [51, 52].

Also, according to the findings, substance abuse media literacy had a significant inverse association with a positive attitude toward drug use. In line with the results of the present study Banerjee et al., Showed that the acquisition of media literacy skills is associated with a negative attitude towards smoking in students [53].

Another result of the present study was the inverse association between substance abuse media literacy skills and subjective norms. Consistent with this result, the findings of Austin et al.’s study showed that young people reduced their beliefs about their peers who smoked by acquiring media literacy skills [54]. On the other hand, in the study of Vahedi et al., the role of acquiring media literacy skills in reducing behavioral intention and performing high-risk behaviors such as drug use and high-risk sexual behaviors was mentioned [19]. Peer pressure seems to play a determinant role in the onset of substance use, and this issue, on the one hand, is due to friendship with peers with drug abuse, and on the other hand, the need to belong to the group in adolescents and young people. Also, results of studies proved life skills training such as refusing skills, media literacy skills education, and critical thinking increase cognitive coping skills and reduce the willingness of high-risk behaviors in youth [8, 41]. Therefore, designing and performing educational and promotional intervention studies in the field of media literacy skills to promote health-oriented behaviors and reduce high-risk behaviors, especially in youth, is recommended.

The important findings of the present study were, based on the EPWM, the variable of behavioral willingness and the variable of behavioral intention were considered the best predictors for the variable of drug use, and behavioral willingness and positive attitude toward drug use were the best predictors for the behavioral intention variable. In general, the results of path coefficients indicate the intensity of the effect of the behavioral willingness variable on the behavior variable more than the behavioral intention variable on this behavior. In other words, the social reaction path had a higher impact on the drug use behavior than the rational path among youth students which is consistent with the results of various studies [27, 28, 55].

This finding indicates that participants are more influenced by emotions and social influence and decide to engage in risky behaviors. Therefore, acquiring skills to increase the capacity of individuals against social influence leads to a decrease in their susceptibility to social influences [56].

Therefore, the design and implementation of educational and promotional programs to reduce behavioral willingness and increase knowledge of the consequences of drug use are necessary to reduce drug use and behavioral intention in students.

This study has several limitations. First, since this was a cross-sectional study, identifying additional factors in future research was recommended. Second, future research should analyze data from a total of Iranian students. At the same time, the potential for interviewer biases may be included, and which longitude study design could help manage bias. However, the findings of this study might not be generalized to all populations of students. Therefore, future research can investigate other factors influencing substance use in youth.

## Conclusion

A qualitative study exploring youth’s willingness to engage in drug abuse can reveal important factors, which in the future research was suggested. In general, the extended prototype willingness model can predict drug use in students. The study’s results showed the importance of the social effects and reactions path instead of the logical, rational action path to investigate this high-risk behavior in students. Therefore, designing and implementing educational and promotional intervention programs to reduce behavioral willingness and increase students’ knowledge of the consequences of drug use is necessary to prevent and reduce drug use behavior. Future studies may consider different constructs from other theoretical models to predict drug use in youth. Also, to design future promotional studies, it is suggested to pay attention to areas such as awareness of the effects and consequences of easy and attractive as the new form of using drugs, acquire specific media literacy; substance abuse media literacy and familiarity with media consumption regime for young people could be helpful.

## Data Availability

All data generated or analyzed during this study are not publicly available to maintain the privacy of the individuals’ identities. The dataset supporting the conclusions is available upon request to the corresponding author.

## Abbreviations

EPWM: Extended prototype willingness model
SMAL: Substance abuse media literacy
PWM: Prototype willingness model
PLS: Partial least squares’ confirmatory composite approach
AVE: Average variance extracted
